# Supplementary material for: Erectile dysfunction after COVID-19 recovery: A follow-up study
Source: PLoS One. 2022 Oct 20;17(10):e0276429. doi: 10.1371/journal.pone.0276429 (PMC9584530; doi:10.1371/journal.pone.0276429)
Supplement: Attachment — Submitted filename: Response to Reviewers.docx [file pone.0276429.s002.docx]

**Response to Editor and Reviewers**

Dear Editor and Reviewers,

We appreciate all your kind comments and suggestions. Hereby, please find our point-by-point responses listed below.

Regards,

On behalf of all authors,

Kavirach Tantiwongse

October 4, 2022

Journal requirements:

Please review your reference list to ensure that it is complete and correct. If you have cited papers that have been retracted, please include the rationale for doing so in the manuscript text, or remove these references and replace them with relevant current references. Any changes to the reference list should be mentioned in the rebuttal letter that accompanies your revised manuscript. If you need to cite a retracted article, indicate the article’s retracted status in the References list and also include a citation and full reference for the retraction notice.

Response: All references have been reviewed, and none of them has been retracted.

Additional Editor Comments:

* Abstract Method; please briefly introduce the main methods that was used in the current study: mixed-effect model and logistic regression model.
Response: Thank you for this important point. We have now mentioned both generalized linear mixed-effect model (GLMM) and logistic regression, the main methods of our study, in the abstract, according to your suggestion. (Page 2 Line 35-38)

* Abstract Results; This section should be in line with the methods section. That is, when method A is introduced in the Method section, the results from this method also should be described in Results section.

Response: Thank you for your helpful advice. Additional results from GLMM have been added. (Page 2 Line 39-44)

* Abstract; If the word count exceeds, introduction section could be simplified, just stating "The present study aimed to ~".

Response: Regarding all changes that have been made, the total word count is 287 for the abstract.
